# Unexpected loss of contact allergy to aluminium induced by vaccine

**DOI:** 10.1111/cod.12053

**Published:** 2013-05

**Authors:** Anette Gente Lidholm, Elisabet Bergfors, Annica Inerot, Ulla Blomgren, Martin Gillstedt, Birger Trollfors

**Affiliations:** 1 Department of Dermatology and Venereology, Sahlgrenska University HospitalS-413 45 Gothenburg, Sweden; 2 The Research and Development Unit in Local Health Care, County of ÖstergötlandS-581 85 Linköping, Sweden; 3 Department of Paediatrics, Sahlgrenska University HospitalS-416 85 Gothenburg, Sweden

**Keywords:** aluminium, contact allergy, itchy nodules, patch test, vaccines

## Abstract

**Background:**

In studies in Gothenburg, Sweden, in the 1990s of an aluminium hydroxide-adsorbed pertussis toxoid vaccine, 745 of ∼76 000 vaccinated children developed long-lasting itchy subcutaneous nodules at the vaccination site. Of 495 children with itchy nodules patch tested for aluminium allergy, 376 (76%) were positive.

**Objectives:**

To study the prognosis of the vaccine-induced aluminium allergy.

**Patients and methods:**

Two hundred and forty-one children with demonstrated aluminium allergy in the previous study were patch tested again 5–9 years after the initial test, with the same procedure as used previously.

**Results:**

Contact allergy to aluminium was no longer demonstrable in 186 of the retested 241 children (77%). A negative test result was more common in children who no longer had itching at the vaccination site; it was also related to the age of the child, the time after the first aluminium-adsorbed vaccine dose, and the strength of the reaction in the first test.

**Conclusions:**

Patch test reactivity to aluminium seems to disappear or weaken with time.

Aluminium-adsorbed vaccines can cause intensely itchy subcutaneous nodules (granulomas) at the injection site. The itch can persist for months to many years. Local hypertrichosis, hyperpigmentation/hypopigmentation and eczema are frequently seen in the itchy area. Persistent itchy nodules have been reported after administration of diphtheria–tetanus–acellular pertussis (DTaP) (alone or in combination), hepatitis B and human papilloma virus vaccines (1–8). The itchy nodules are often related to contact allergy to aluminium as shown by a patch test (7–12). As aluminium is present in many vaccines, antiperspirants, and dermatological products, aluminium allergy with contact dermatitis after aluminium exposure is not a trivial problem.

In studies performed in Gothenburg, Sweden in the 1990s of an acellular pertussis vaccine from Statens Serum Institute (SSI), Copenhagen, Denmark, an unexpectedly high frequency of persistent itchy nodules occurred. A total of 645 cases in ∼76 000 vaccinated children were found (7). The children had suffered from chronic or intermittent, often intense, local itching for several months to 10 years. At the time of the first publication, 75% had not yet recovered, so the final outcome could not be predicted (7). After the initial publication of 645 cases of itchy nodules, another 100 cases among children who had participated in the same trials were found, resulting in a total of 745 cases of itchy nodules among 76 000 recipients of the vaccine from SSI. These last 100 cases have not been published previously. In the previous publication (7), 455 children were patch tested for aluminium allergy. Of those, 352 were positive. In a control group, 211 children (siblings of the former group) who received the same aluminium hydroxide-containing vaccines without itchy nodules were tested. Surprisingly, 17 (8%) were positive (*p* < 0.0001) (7). In contrast, none of the 54 siblings who were vaccinated with aluminium phosphate-containing diphtheria–tetanus (DT) vaccine or DTaP from producers other than SSI had a positive patch test reaction.

There are only sparse data on the prognosis of children with vaccine-related itchy nodules and children with confirmed aluminium allergy.

The aim of the present study was to investigate the prognosis of contact allergy to aluminium in children caused by vaccination with aluminium-adsorbed vaccines by repeating the patch test when ≥5 years had elapsed after the initial test.

## Patients and Methods

The present study was a follow-up of children with aluminium allergy related to vaccination with aluminium hydroxide-adsorbed DT vaccine alone, acellular pertussis (aP) vaccine alone or DTaP vaccine combined with other vaccines from SSI (7).

The children/adolescents or their parents were asked to participate in the present study if all of the following criteria were fulfilled: (i) they had current or previous vaccine-related itching nodules after administration of a vaccine from SSI; (ii) they had previously been tested with a patch test and found to be positive for aluminium allergy; (iii) more than 5 years had elapsed since the previous test; and (iv) they were still residing in the Gothenburg area.

In the initial report, 645 children with itching nodules were identified. Of those, 455 were tested and 352 were positive (7). After this report, another 100 children with persistent itching nodules at the vaccination site were identified. Of those, 40 had been patch tested for aluminium allergy and 25 were positive.

**Fig. 1:**
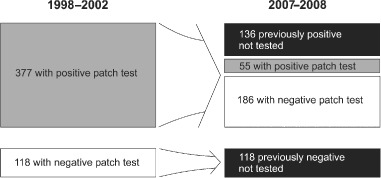
Overview of individuals tested and their test results in 2007–2008.

Among the 377 previously positive patients, 363 children fulfilled the inclusion criteria and were offered renewed testing. The parents of 92 children did not answer, and 16 declined participation; 9 children did not show up on the offered test date. Of the remaining 246 children, 4 came for application of the test but not for the reading, and 1 was not tested because of generalized, severe itching following widespread tattooing with aluminium-containing material. Thus, in all, 241 children were tested with evaluable test results. Of those, 236 had been included in the first report of 645 children with itching nodules (7), and 5 were among the 100 who were discovered after that publication (Fig. 1).

The initial tests were performed between February 1998 and October 2002. The tests reported here were performed between October 2007 and May 2008.

The age of the 241 participants (89 boys; 152 girls) in the present study at the time of retesting varied between 8 and 21 years (median 13.3 years); see Table 1.

**Table 1:** Demographics and patch test results: 2007–2008

	Number of patients (%)	Age at first SSI dose (years), median (min.–max.)	Time from first SSI dose to test (years), median (min.–max.)	Age at test (years), median (min.–max.)
Total	241	1.1 (0.0–9.1)	11.9 (8.6–16.0)	13.3 (8.9–21.2)
Sex				
Male	89 (36.9)	1.0 (0.0–7.9)	11.7 (8.6–15.4)	12.8 (8.9–18.9)
Female	152 (63.1)	1.3 (0.2–9.1)	11.9 (9.0–16.0)	13.6 (10.0–21.2)
Patch test result				
Positive	55 (22.8)	0.3 (0.0–8.1)	11.4 (8.6–12.5)	11.9 (8.9–18.9)
Negative	186 (77.2)	1.3 (0.2–9.1)	11.9 (8.6–16.0)	13.5 (8.9–21.2)

SSI, Statens Serum Institute.

All children but 7 had received a new aP vaccine consisting of 40 µg of pertussis toxoid alone or combined with diphtheria and tetanus toxoids during infancy or childhood (13, 14). Only 6 children had received DT vaccine alone (13), and 1 child had received a DTaP plus inactivated polio plus *Haemophilus influenzae*, type b (Hib) vaccine (15). All vaccines contained aluminium hydroxide corresponding to 0.5 mg of aluminium per dose, except the DTaP–polio–Hib vaccine, which contained 1 mg of aluminium per dose. Details on the numbers of vaccine doses, age at vaccination and vaccination schedules have been published elsewhere (7).

The patch test was applied on the upper part of the back with aluminium in two forms: metallic aluminium (empty Finn Chamber®; SmartPractice, Phoenix, AZ, USA), and aluminium salt (aluminium chloride hexahydrate 2% in petrolatum; Chemotechnique Diagnostics®, Vellinge, Sweden). The aluminium salt was placed in a plastic chamber (16). All applications were performed by one of the authors (U.B.), who has special training and experience. The patches were removed by the parents after 48 hr, and were read another 24 hr later by one of the two dermatologists among the authors (A.G.L. and A.I.). The results were interpreted according to the recommendations of the International Contact Dermatitis Research Group. Positive reactions from + to +++ were regarded as delayed (type IV) hypersensitivity. If the results for aluminium in pet. and metallic aluminium differed between positive and negative and in the strength of the reaction, the highest score was always used. In 27 cases, the result of the aluminium testing did not fulfil the criteria for a + reaction. These doubtful cases have been classified as negative.

Before the test was applied, all parents and children were interviewed regarding ongoing local itching, or duration of the itching if it had stopped. The vaccination site was inspected and examined for remaining nodules. The results of the interviews and the clinical examinations were not known by the reading dermatologist.

Furthermore, parents were asked about exposure to aluminium-containing products (notably antiperspirants, sunscreen products, and aluminium-adsorbed vaccines). The information obtained was usually not highly reliable, because the parents often did not know which vaccines the children had received and whether the other products contained aluminium or not. These results are therefore not reported here.

### Statistical methods

All data were analysed with r version 2.10.1 (R Foundation for Statistical Computing, Vienna, Austria).

Multivariate logistic regression was performed with negative patch test result as the response variable, and time from the first SSI dose, age at the first SSI dose and itching as predictors.

Fisher's exact test was used for contingency tables. The exact binomial version of McNemar's test was used to test for differences in proportions of positive patients in previous tests and the tests in this study. The exact permutation version of Wilcoxon's rank sum test was used to test for differences in ages and time from first SSI dose between groups.

All tests are two-sided, and *p* < 0.05 was considered to be statistically significant.

### Ethical considerations

The study was approved by the Regional Ethical Review Board of Gothenburg University. All parents, adolescents and young adults received oral and written information about the study, and all parents and/or young adults gave written consent to participate.

## Results

Of the 241 children who came both for the application of the test and for the reading, 186 [77.2%, 95% confidence interval (CI) 71.4–82.3%] had negative test results, and 55 (22.8, 95% CI 17.7–28.6%) still had positive test results.

In total, 495 patients had been tested earlier (455 in the previous study and 40 shortly after the publication; see above). Of the 495 patients tested, 377 (76.2%) had a positive patch test result. Of those 377 positive patients, 241 were tested in this study. Hence, the status of the 136 missing patients with previous positive patch test results and the 118 with previous negative results is unknown. On the assumption that all of these 254 patients with unknown status would have been positive or negative had they been tested in this study, the proportion of positive patients of the original 495 could be anywhere between 11.1% and 62.4%. In the worst case scenario, the proportion with positive patch test results would have decreased from 76.2% in the previous study to 62.4% in the current study, which is still a significant decrease (*p* = 0.00011, exact McNemar's test). Our most realistic estimate however, would be a decrease from 76.2% to 17.4%, assuming that the 136 previously positive missing cases were randomly distributed and the 118 previously negative cases remained negative.

**Fig. 2:**
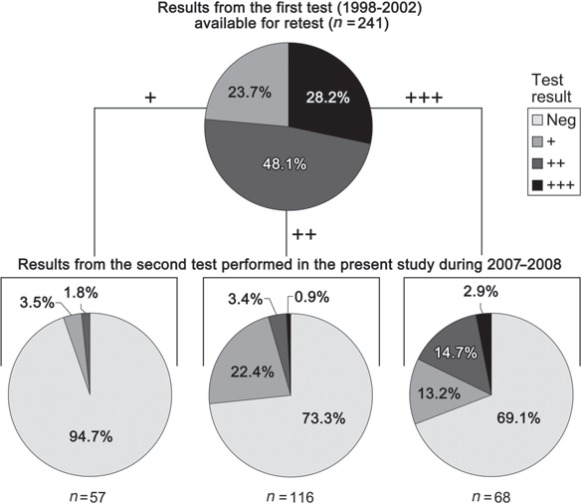
Results of the aluminium test in the second test performed in the present study during 2007–2008 in relation to the results of the first test in the same individuals performed in 1998–2002 (7).

Of the 241 children tested a second time, 186 (77.2%) were negative, 37 (15.4%) had a weak (+) reaction, 15 (6.2%) had a ++ reaction, and 3 (1.2%) had the strongest (+++) reaction. Of those who scored ++ or +++ on the first test, 71.7% (95% CI 64.6–78.1%) were negative on the second test. Of those who scored + on the first test, 94.7% (95% CI 85.4–98.9%) were negative on the second test, which is significantly more (*p* = 0.00012) than those with ++ or +++ reactions (Fig. 2).

Of all 241 retested children, 66 had ongoing local itching at the site of vaccination at the time of testing. Of those 66, 22 had positive patch test results (33.3%, 95% CI 22.2–46.0%). Of the 175 children who no longer had local itching, 33 (18.9%, 95% CI 13.4–25.5%) had positive patch test results, which is a significantly lower proportion (*p* = 0.025, Fisher's exact test).

Only 5 children had palpable subcutaneous nodules at the vaccination site. All of them still had itching, but all were negative in the test. The number was too small for statistical calculations.

The median age of the children with positive patch test results was 11.9 years (range 8.9–18.9 years), and that of the children with negative patch test results was 13.5 years (range 8.9–21.2 years), which is significantly higher (*p* = 0.0002, Wilcoxon's rank sum test).

The median time between the first vaccination and the second patch test described here was 11.4 years (range 8.6–12.5 years) for those who were still positive, and that for those who had become negative in the second test was 11.9 years (8.6–16 years), which is significantly higher (*p* < 0.0001, Wilcoxon's rank sum test).

**Fig. 3:**
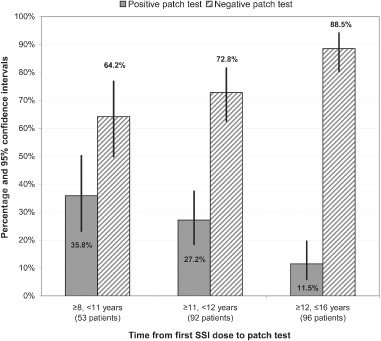
Patch test results in the retest study, as a function of time from the first Statens Serum Institute (SSI) dose, with the children divided into groups with different times elapsed since their first SSI vaccine.

A multivariate analysis (Table 2) showed that the probability of having a negative patch test result was positively correlated with the time from the first dose of vaccine produced by SSI (*p* = 0.001). The proportion of negative cases among those who were tested within 11 years from the first SSI dose was 64.2% (95% CI 49.8–76.9%). Among those tested ≥ 12 years from the first SSI dose, 88.5% were negative (95% CI 80.4–94.1%); see Fig. 3.

**Table 2:** Multivariate logistic regression

Predictor	Coefficient estimate	Coefficient standard error	*p*-value
Intercept	−6.04	2.04	—
Time from first SSI dose to patch test (years)	0.57	0.18	0.0010
Age at first SSI dose (years)	0.15	0.11	0.15
Itching [yes (reference) versus no]	0.76	0.34	0.027

SSI, Statens Serum Institute.

The probability of a negative patch test result is the response variable.

The children who no longer had itching were more likely to have a negative patch test result than those who still had itching (*p* = 0.027).

The age of the patient at the time of the first SSI dose did not significantly affect the patch test outcome when the other two above-mentioned variables were included in the analysis.

## Discussion

Contact allergy to >4000 substances has been described (17), and the allergy has been considered to persist throughout life (18). However, there are only a few modern studies addressing this issue. Lee and Maibach in 2001 (19) published a review article on patch test follow-ups years after the initial test, under the heading: ‘Is contact allergy in man life long?’ They found four articles on different separate substances, colophonium (20), nickel (21, 22), and cobalt (23). The number of retested persons varied from 50 to 104 in the different studies. The proportion who had become negative in the test ranged from 4% to 23%. Josefson et al. (24) retested 369 women in Sweden more than 20 years after the initial study. Of 38 schoolgirls positive for nickel in the early 1980s, 28 were verified to be positive at retesting, indicating that 30% had become negative. In a study from 1954 (25), dermatology patients in Denmark were patch tested 2–19 years after the first occasion, and it was found that 111 of 188 positive reactions in the standardized test at that time had ‘disappeared’ (59%).

Even if the contact allergy remains, the connected contact dermatitis can heal if the individual is not exposed to the allergen. Some of the most important contact allergens are metals, for example nickel, cobalt, and chromium. Aluminium can be added to this list, although clinically relevant contact dermatitis is very uncommon (26). The most important routes of aluminium exposure are vaccination with aluminium-containing vaccines, hyposensitization with aluminium-adsorbed extracts, and contact with aluminium in antiperspirants and other skin care products.

Ideally, the present study should have had a control group consisting of the children who had vaccine-related itchy nodules with negative patch test results at the initial testing when they were tested with ongoing or recent pruritus for the first time ≥ 5 years ago (7). Informal contacts between one of the authors (B.T.) and members of the regional ethics committee indicated, however, that it would be difficult to obtain permission to retest children who had never had a positive test result. We agreed with that evaluation, and therefore did not offer retesting of initially negative children.

The main finding in the present study was that aluminium allergy as shown by a positive patch test result and related to vaccination with aluminium-containing vaccines had decreased significantly. With the assumptions made above, it had probably ‘disappeared’ in 77% of children with a previous positive test result. In follow-up patch test studies, such a large proportion turning negative in the second test has never been shown before; in contrast, contact allergies are mostly described as being chronic (18, 27). In light of the common opinion among dermatologists and in the major textbooks in the field (27) that aluminium is a very uncommon allergen among unselected children, the rate of positive test results among the children retested here still is very high. The consequence of this is that a large number of children may bring their allergy with them into adolescence and adult life, leading to problems when they need other aluminium-adsorbed vaccines, and when they need, but cannot use, antiperspirants.

We are aware of only one previous study in which children with demonstrated aluminium allergy had been retested (10). In that study from Denmark, 4 patients with a positive patch test result following vaccination with aluminium hydroxide-adsorbed DTaP were retested (interval not stated). Two of them had become negative. The same study showed, in agreement with the present study, that the subcutaneous nodules and the local itching could resolve with time. Of 21 children followed for 1–8 years, 5 healed and 11 improved clinically.

Disappearance of aluminium allergy as measured with the patch test was significantly correlated with disappearance of local itching. The chance of having a negative second test was also higher with increasing age and a longer time interval between the first vaccination and the second test. The numerical difference for the time elapsed between the first vaccine dose and the date of the test was, however, rather small; only 0.5 years between those with a negative test result and a those with a positive test result. In this study, this time interval seems to be of little clinical relevance.

The results must be regarded with some caution, as it has been suggested that a concentration of aluminium chloride hexahydrate of >2% is needed to detect all cases of aluminium allergy (28). In one study of 37 children who had undergone hyposensitization therapy with aluminium-containing extracts, aluminium allergy was shown in 8 patients with 10% aluminium chloride hexahydrate, whereas only 3 were clearly positive when the concentration was 2% (26). There are many publications on aluminium in the literature concerning contact dermatitis, but we have not found a consensus about which aluminium compound, vehicle and concentration could be the gold standard for patch testing. In the handbook commonly used in patch test clinics (17), more than a handful of different aluminium compounds in different vehicles and concentrations are listed. The reason why 2% was used in the previous study (7) and present study was that this concentration is used in the baseline series in patch testing and that it is the only one commercially available. Furthermore, a large number of children reacted very strongly to the 2% solution in the previous study (8), making us hesitant to apply a higher concentration. Besides, there is no consensus regarding what amount of pet. preparation to apply while patch testing. In the present study, one of the authors (U.B.) performed the application of aluminium in pet. and the Finn Chamber®. In the previous study, these applications were performed by a physician (E.B.) or two nurses, who had all been instructed by U.B. on how to perform the applications. Swedish investigators have shown that there is both intraindividual and interindividual variation in the amount of pet. applied, but the individual technician can keep the variation within a limited range (29).

Thus, even though we cannot state that the children/young adults in this study had become free from their aluminium allergy, it is clear that the magnitude of the allergy must have decreased.

The interaction between itching nodules and aluminium allergy is unclear, and cannot be clarified by this study. As vaccinations start at an early age in healthy children, it is hardly conceivable that the children were aluminium-allergic prior to the vaccinations. It is more likely that the sensitization reaction to aluminium has led to the granuloma formation. Sarcoidal-type allergic contact granuloma was first described in 1983 from gold earrings (30) Palladium-induced contact granuloma has also been reported (31). Case reports of injected aluminium in a tattoo or cosmetic eyebrows have been described (32, 33). Histopathological examination of the nodules has shown granuloma formations in which aluminium can be shown by staining and atomic absorption spectrometry (5, 6, 34).

In conclusion, the present study showed that previously diagnosed aluminium allergy in 241 children following vaccination with aluminium-adsorbed vaccines could be shown in only 55 (22.8%) when ≥5 years had elapsed after the initial testing, with the same procedure and a concentration of 2% aluminium chloride hexahydrate in the patch test. Disappearance of aluminium allergy as measured by the patch test was significantly correlated with disappearance of local itching. The chance of having a negative second test was also higher with increasing age and a longer time interval between the first vaccination and the second test. The numerical difference for the time elapsed between the first vaccine dose and the date of the test was, however, rather small, at only 0.5 years between those with a negative patch test result and those with a positive patch test result. In this study, this time interval seems to be of little clinical relevance. Furthermore, the likelihood of having a negative patch test result at the second test was significantly higher if the intensity of the positive patch test result in the first study was low (+) than if it was high (++ or +++).
